# MiR-34b-5p Suppresses Melanoma Differentiation-Associated Gene 5 (*MDA5*) Signaling Pathway to Promote Avian Leukosis Virus Subgroup J (ALV-J)-Infected Cells Proliferaction and ALV-J Replication

**DOI:** 10.3389/fcimb.2017.00017

**Published:** 2017-01-30

**Authors:** Zhenhui Li, Qingbin Luo, Haiping Xu, Ming Zheng, Bahareldin Ali Abdalla, Min Feng, Bolin Cai, Xiaocui Zhang, Qinghua Nie, Xiquan Zhang

**Affiliations:** ^1^Department of Animal Genetics, Breeding and Reproduction, College of Animal Science, South China Agricultural UniversityGuangzhou, China; ^2^Guangdong Provincial Key Lab of Agro-Animal Genomics and Molecular Breeding and the Key Lab of Chicken Genetics, Breeding and Reproduction, Ministry of AgricultureGuangzhou, China

**Keywords:** MiR-34b-5p, melanoma differentiation-associated gene 5 (MDA5), Avian leukosis virus subgroup J (ALV-J), Cell proliferation, MDA5 signaling pathway

## Abstract

Avian leukosis virus subgroup J (ALV-J) is an oncogenic retrovirus that has a similar replication cycle to multiple viruses and therefore can be used as a model system for viral entry into host cells. However, there are few reports on the genes or microRNAs (miRNAs) that are responsible for the replication of ALV-J. Our previous miRNA and RNA sequencing data showed that the expression of miR-34b-5p was significantly upregulated in ALV-J-infected chicken spleens compared to non-infected chicken spleens, but melanoma differentiation-associated gene 5 (*MDA5*) had the opposite expression pattern. In this study, a dual-luciferase reporter assay showed that *MDA5* is a direct target of miR-34b-5p. *In vitro*, overexpression of miR-34b-5p accelerated the proliferation of ALV-J-infected cells by inducing the progression from G2 to S phase and it promoted cell migration. Ectopic expression of *MDA5* inhibited ALV-J-infected cell proliferation, the cell cycle and cell migration, and knockdown of *MDA5* promoted proliferation, the cell cycle and migration. In addition, during ALV-J infections, *MDA5* can detect virus invasion and it triggers the MDA5 signaling pathway. *MDA5* overexpression can activate the MDA5 signaling pathway, and thus it can inhibit the mRNA and protein expression of the ALV-J *env* gene and it can suppress virion secretion. In contrast, in response to the knockdown of *MDA5* by small interfering RNA (siRNA) or an miR-34b-5p mimic, genes in the MDA5 signaling pathway were significantly downregulated (*P* < 0.05), but the mRNA and protein expression of ALV-J *env* and the sample-to-positive ratio of virion in the supernatants were increased. This indicates that miR-34b-5p is able to trigger the MDA5 signaling pathway and affect ALV-J infections. Together, these results suggest that miR-34b-5p targets *MDA5* to accelerate the proliferation and migration of ALV-J-infected cells, and it promotes ALV-J replication, via the MDA5 signaling pathway.

## Introduction

Avian leukosis virus subgroup J (ALV-J) is a novel, virulent subtype in the avian leukosis virus family (ALVs), which can be classified into 10 subgroups (Payne, 1998). In birds, ALV-J can induce myeloid leukosis, various tumors, growth retardation, and serious immunosuppression (Stedman and Brown, 1999; Payne and Nair, 2012; Zeng et al., 2014). There is no effective vaccine against ALV-J (Payne and Nair, 2012). Furthermore, as an oncogenic retrovirus, ALV-J shares a similar replication cycle with a number of RNA and DNA viruses, such as human immunodeficiency virus type-1 (HIV-1), which can replicate in both the nucleus and the cytoplasm. Therefore, it can be used as a model system for viral entry into host cells. Thus, identifying the pathogenic mechanism of ALV-J can provide a solid foundation for further study of other retroviruses. Recently, several genes and the extracellular signal-regulated kinase (ERK)/ mitogen-activated protein kinase (MAPK) pathway have been demonstrated to be important for ALV-J replication and oncogenesis (Chai and Bates, 2006; Li Y. et al., 2014; Dai et al., 2016). However, the microRNAs (miRNAs) and pathways involved in ALV-J infections largely remain unclear.

MiRNAs are small non-coding RNAs that play important roles in cell growth, proliferation, and apoptosis by inducing translational inhibition or mRNA degradation (Sun et al., 2010; Feng et al., 2011). Aberrant expression of miRNAs has been linked to tumorigenesis (Zhao et al., 2009; Datta et al., 2012). To date, the expression of several miRNAs has been found to change in response to ALV-J infection. For example, four miRNAs (gga-miR-125b, gga-miR-193a, gga-miR-193b, and gga-miR-221) that are known to be involved in tumorigenesis-related pathways were shown to be expressed abnormally in ALV-J-infected chickens, and they might be associated with ALV-J-induced tumorigenesis (Wang et al., 2013a). It has been shown that the binding of gga-miR-1650 to several sites in the 5′ untranslated region (UTR) of ALV-J genomic RNA is critical for viral replication (Wang et al., 2013b). In addition, gga-miR-221, gga-miR-222, and gga-miR-375 have been shown to play a pivotal role in tumorigenesis after an ALV-J infection (Li H. et al., 2014; Dai et al., 2015).

We previously showed that miRNA-23b promotes ALV-J replication by targeting interferon regulatory factor-1 (*IRF1*; Li et al., 2015). The miRNA sequencing data (Gene Expression Omnibus; GEO database, accession number GSE63676) also showed that another miRNA, gga-miR-34b-5p, was upregulated in ALV-J-infected chickens compared to non-infected chickens, suggesting that this miRNA might also be involved in ALV-J infections. In humans, miR-34b has been reported to be involved in many neoplastic diseases and low levels of expression of miR-34b were found to be associated with gastric tumorigenesis (Tsai et al., 2011).

Vertebrate innate immune responses are mediated by pattern recognition receptors (PRRs), including Toll-like receptors (TLRs), retinoic acid-inducible gene I-like receptors (RLRs), and Nod-like receptors (NLRs). *MDA5* is a member of the RLR family, which are comprised of N-terminal tandem caspase activation and recruitment domains (CARDs), a central helicase domain responsible for RNA-dependent ATP hydrolysis and a C-terminal regulatory domain (CTD; Kato et al., 2006). Moreover, *MDA5* can activate the interferon (IFN) signaling pathway and it thereby plays a critical role in antiviral innate immunity. Our previously published RNA sequencing data (GSE63226) showed that *MDA5* was downregulated in ALV-J-infected chickens compared to non-infected chickens. However, relatively little is known regarding the effect of ectopic expression of *MDA5* in ALV-J-infected chickens.

The first aim of this study was to explore whether or not miR-34b-5p is involved in ALV-J infections and to clarify how it affects ALV-J replication, as well as to characterize the oncogenesis in chicken fibroblast cell line (DF-1) infected with ALV-J. We confirmed that miR-34b-5p was increased in ALV-J-infected tissues and that ectopic expression of miR-34b-5p accelerated the proliferation and migration of ALV-J-infected cells. *MDA5* was verified as a direct target of miR-34b-5p. Further *in vitro* investigations demonstrated that miR-34b-5p can regulate the MDA5 signaling pathway, the expression of the ALV-J *Env* gene, and virion secretion. Taken together, these results suggest that miR-34b-5p accelerates the proliferation and migration ALV-J-infected cells and promotes ALV-J replication by targeting *MDA5*.

## Materials and methods

### Ethics statement

All animal experiments in this study were performed following protocols approved by the Institutional Animal Care and Use Committee at the South China Agricultural University, China (approval ID: SCAU#0011). All the experiments were carried out in compliance with the regulations and guidelines established by this committee. The authors declare that all animal experiments under the consent of the animal owners. All efforts were made to minimize suffering to animal. The authors declare no additional considerations of the study in cases where vulnerable populations were involved.

### Animals

Two chicken groups, ALV-J-infected broilers and non-infected broilers at 140 days old, were used. The animals were the same as those used in our previous RNA sequencing study (Li et al., 2015).

### Virus propagation and quantification of virus titer

SCAU-HN06, a strain of ALV-J, was kindly provided by Assoc. Prof. Weisheng Cao (South China Agricultural University). The chicken embryonic fibroblast cell line (DF-1 cells) was obtained from the Cell Bank of the Committee on Type Culture Collection of the Chinese Academy of Sciences and it was cultivated adherently in cell culture vessels containing Dulbecco's Modified Eagle's Medium (DMEM; Gibco, Life Technologies, Grand Island, NY, USA) supplemented with 10% fetal bovine serum (FBS; Gibco, Life Technologies, Grand Island, NY, USA) at 37°C in an atmosphere of 5% CO_2_. When the cells grew to a density of 80% confluence, they were infected with the ALV-J strain SCAU-HN06. The inoculum was discarded 2 h later and it was replaced with DMEM containing 1% FBS, 100 U/mL penicillin, and 100 ug/mL streptomycin. After incubation for 6 days in culture, the cell supernatants (which contained the viral particles) were harvested as described previously (Dai et al., 2016). The virus was diluted 10-fold using serial dilution techniques in DMEM and the suspension was then applied to DF-1 cells for 6 days in a 96-well plate. The virus was titrated to determine the 50% tissue culture infectious doses (TCID_50_) in accordance with the method described by Reed and Muench (1938).

### RNA isolation and cDNA synthesis

Total RNA was extracted from the tissue samples and DF-1 cells using TRIzol® as recommended by the supplier (Invitrogen Life Technologies, Carlsbad, CA, USA). The integrity and quantity of RNA were assessed using 1.5% agarose gel electrophoresis and spectrophotometry (ND-2000, NanoDrop Inc., Wilmington, DE, USA), respectively. A PrimeScript RT Reagent Kit (Takara, Otsu, Japan) was used to carry out cDNA synthesis of mRNA, following the manufacture's protocol. The synthesized cDNA was stored at −20°C until subsequent analysis using real-time quantitative polymerase chain reaction (RT-qPCR).

### Primers

RT-qPCR primers that were specific for *MDA5*, ALV-J *Env, Gag, Pol, Mx1*, and β*-actin* were designed using Premier Primer 5.0 software. RT-qPCR primers that were specific for genes in the MDA5 signaling pathway, including interferon-β promoter stimulator 1 (*IPS-1*), interferon regulatory factor-3 (*IRF-3*), interferon-β (*IFN*β), double-stranded RNA-dependent protein kinase (*PKR*), 2′, 5′-oligoadenylate synthetase (*OAS*), myxovirus (influenza virus) resistance 1, interferon-inducible protein p78 (mouse) (*Mx1*), and major histocompatibility complex class I (*MHC class I*), are referenced in previous publications (Supplementary Table 1; Lee C. C. et al., 2014). PCR primers for the full-length *MDA5* coding sequence clone and the 3′ UTR of the *MDA5* clone were also designed using the Premier Primer 5.0 software (Supplementary Table 2). All the above primers were synthesized by Sangon Biotech Co., Ltd. (Guangzhou, China). A bulge-loop™ Reverse Transcription primer and RT-qPCR primers that were specific for gga-miR-34b-5p were designed and synthesized by RiboBio (Guangzhou, China).

### RNA oligoribonucleotides and plasmids construction

Gga-miR-34b-5p mimics, mimic control duplexes, small interfering RNA (siRNA) targeted against the *MDA5* gene (si-*MDA5*), and a non-specific siRNA control duplex were designed and synthesized by RiboBio (Guangzhou, China). Oligonucleotide sequences of si-MDA5 were showed in Supplementary Table 3. To construct a gga-miR-34b-5p target luciferase reporter vector (pmirGLO-WT MDA5-3′UTR), the segment sequence of the *MDA5* 3′ UTR (666 bp) that contained the putative gga-miR-34b-5p binding sequence was amplified by PCR using a cDNA template synthesized from total RNA. Subsequently, the PCR product was sub-cloned into NheI/SalI restriction sites in the pmirGLO dual-luciferase reporter vector (Promega, Madison, WI, USA) to generate the pmirGLO- WT-MDA5-3′UTR reporter vector.

However, to generate a gga-miR-34b-5p target-mutated reporter vector (pmirGLO-MT-MDA5-3′UTR), mutations were achieved by changing the gga-miR-34b-5p binding seed sequences from ACTGCCT to GACTATC using the megaprimer PCR method (Ke and Madison, 1997). An *MDA5* overexpression construct was generated by amplifying the *MDA5* coding sequence, and it was subsequently cloned into the overexpression plasmid vector, pSDS-20218, which was purchased from Shanghai SiDanSai Biotechnology Co., Ltd., China (http://www.sidansai.com/).

### Transfection of MDA5 overexpression plasmid, si-MDA5, and miR-34b-5p mimics and preparation of ALV-J

When DF-1 cells grew to a density of 50% confluence, they were transfected with (a) the *MDA5* overexpression plasmid, (b) si-MDA5, or (c) the gga-miR-34b-5p mimic using Lipofectamine 3000 Reagent (Life Technologies, USA), in accordance with the manufacture's recommended protocol., After 12 h, the cells were inoculated with TCID_50_ of ALV-J. After 2 h of incubation, the supernatant was discarded and the infected cells were replenished with DMEM medium containing 1% FBS, 100 U/mL penicillin, and 100 ug/mL streptomycin.

### Luciferase reporter assay

Luciferase activity was measured using Dual-GLO® Luciferase Assay System Kits (Promega, Madison, WI, USA) following the manufacturer's instructions. DF-1 cells were seeded at a density of 1 × 10^3^ cells per well in 96-well plates. After 24 h, the cells were co-transfected with 100 ng pmir-GLO- WT-MDA5-3′ UTR- (wild-type) or pmir-GLO-MT-MDA5-3′ UTR(mutant-type) plasmids, or 100 nM gga-miR-34b-5p mimic and miR-NC using the Lipofectamine 3000 Reagent. Fourty-eight hours after transfection, luciferase assays were performed using a Fluorescence/Multi-Detection Microplate Reader (Synergy 2, Biotek, Winooski, VT, USA). The values obtained were normalized to the levels of a Renilla luciferase plasmid (pRL-TK Vector) levels.

### RT-qPCR analysis

The RT-qPCR reactions were performed using a Bio-Rad CFX96 Real-Time PCR Detection System using iTaq™ Universal SYBR® Green Supermix Kits (Bio-Rad Laboratories Inc., Hercules, CA, USA) to detect the mRNA expression level associated with each gene. The chicken β*-actin* gene was used as an internal control. The analysis was carried out using the 2^−ΔΔCt^ method, as described previously (Livak and Schmittgen, 2001).

### Wound healing assay

We also carried out a wound healing migration assay. First, DF-1 cells were seeded in 12-well plates. When the cells grew to a density of 50% confluence, they were transfected with the *MDA5* overexpression plasmid, si-MDA5, or a gga-miR-34b-5p mimic, and then they were infected with ALV-J, as described above. When the cells reached a density of 100% confluence, a linear wound was generated by scratching the monolayer of cells with a 1 mL pipette tip. The cells that migrated into the wound area were captured at 0 (control), 24, 48, and 72 h using a microscope (Nikon, Shinagawa, Tokyo, Japan). Width of the scratches was measured using the Image Pro Plus 6.0 software (Media Cybernetics). The data of each scratch width were presented in Supplementary Table 4. The formula to calculate the cell migration rate was as follows: (W_0h_ − W_xh_) × 100%/W_0h_, where W_0h_, represents the mean wound width at 0 h and W_xh_ represents the mean wound width at 24, 48, and 72 h.

### Flow cytometry analysis

For the flow cytometry analysis of the cell cycle, DF-1 cells were seeded in 12-well plates. When the cells grew to a density of 50% confluence, they were transfected and then infected with ALV-J, as described above. After being infected, the cells were incubated for 72 h, collected and fixed overnight with 75% ethanol at 4°C. The fixed cells were stained with 50 μg/mL propidium iodide solution (Sigma Life Science, St. Louis, MO, USA) containing 10 μg/mL RNase A (Takare) and 0.2% (v/v) Triton X-100 (Sigma), and they were incubated in the dark for 30 min at 37°C. The cell cycle phase analysis of the incubated cells was performed using a BD Accuri C6 flow cytometer (BD, San Jose, CA, USA).

### Sodium dodecyl sulfate polyacrylamide gel electrophoresis (SDS-PAGE) and western blotting

SDS-PAGE and Western blotting were performed using methods that have been described in detail previously (Feng et al., 2016). The primary antibodies used were as follows: ALV-J-specific monoclonal antibody JE-9 (which was kindly provided by Prof. Aijian Qin, Yangzhou University, China) and mouse polyclonal anti-glyceraldehyde 3-phosphate dehydrogenase (anti-GAPDH; Bioworld Technology, Inc., USA). Goat anti-rabbit IgG (heavy and light chains (H+L))-horseradish peroxidase (HRP) and goat anti-mouse IgG (H+L)-HRP (Bioworld Technology, Inc., USA) served as secondary antibodies. The DF-1 cells were seeded in 6-well plates. When the cells grew to a density of 50% confluence, they were transfected, and then infected with ALV-J, as described above. After 3 days in culture, the DF-1 cells were subjected to the SDS-PAGE and Western blot analyses. Detection of HRP-conjugated secondary antibodies was performed using Western Lightning Chemiluminescence Reagent Plus (Perkin Elmer Life Sciences, Inc, Boston, MA, USA), and images were developed on X-ray films.

### Enzyme-linked immunosorbent assay (ELISA)

For the ELISA, the DF-1 cells were seeded in 96-well plates. When the cells grew to a density of 50% confluence, they were transfected, and then infected with ALV-J, as described above. At the indicated times (24, 48, 72, 96, and 120 h), the supernatants were collected. After three repetitions of freezing and thawing the supernatants, the ALV-J p27 protein expression level was measured using an ELISA ALV-J diagnosis kit, as described in the manufacturer's protocol (IDEXX, USA).

### Cell-counting kit-8 (CCK-8) assay

For the cell growth assays, DF-1 cells were seeded in 96-well plates. When the cells grew to a density of 50% confluence, they were transfected, and then infected with ALV-J, as described above. The viable cells were detected every 24 h using a CKK-8 (Dojindo, Kumamoto, Japan), in accordance with the manufacturer's protocol. Absorbance was measured using a Model 680 Microplate Reader (Bio-Rad, Hercules, California, USA) by optical density at a wavelength of 450 nm.

### Statistical analysis

Each experiment was performed in triplicate. The data are presented as the mean ± the standard error of the mean (SEM) of each set of three independent experiments. Where applicable, the statistical significance of the data was tested using one-sample or paired *t*-tests. The types of tests and the *P*-values, when applicable, are indicated in the figure legends.

## Results

### MIR-34b-5p is aberrantly upregulated in ALV-J-infected chickens

**Figure 1:**
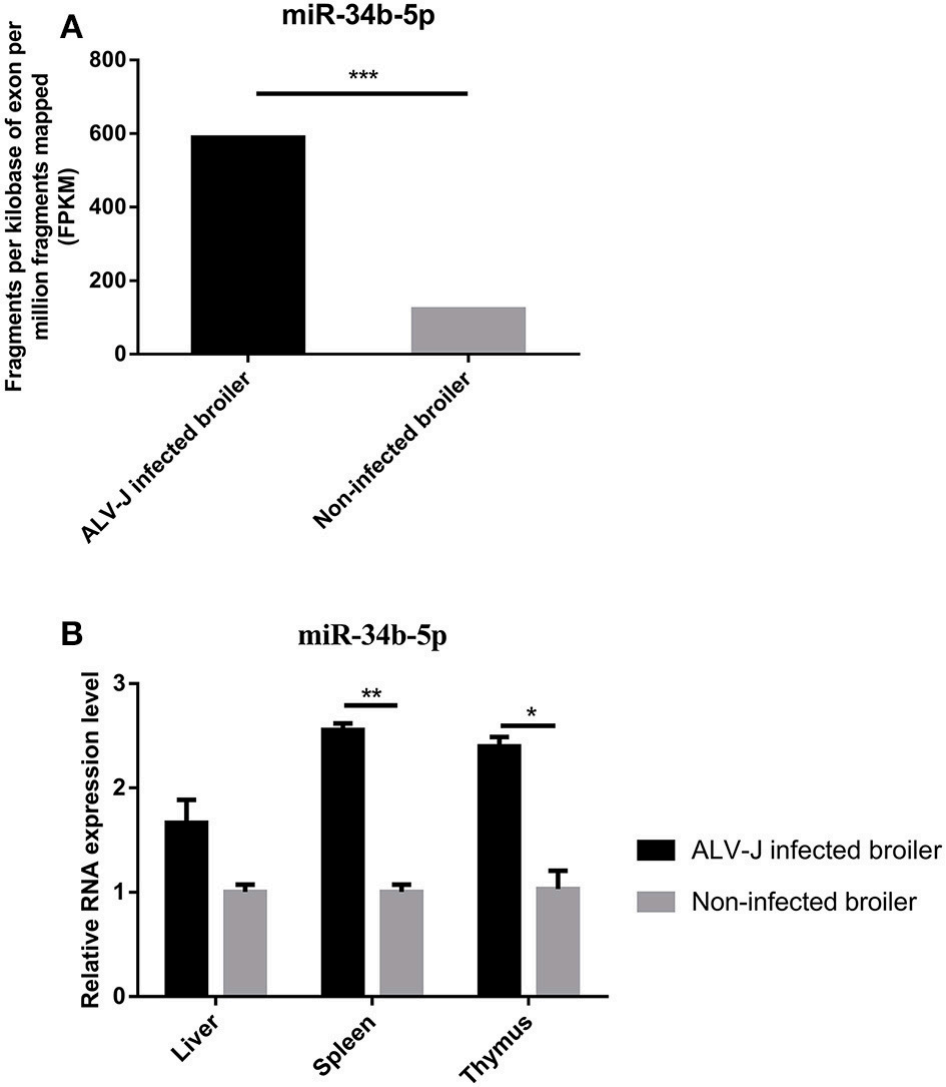
**Relative expression of miR-34b-5p in ALV-J-infected and non-infected tissues. (A)** MiRNA sequencing showed that miR-34b-5p was upregulated in the spleen of ALV-J-infected chickens. **(B)** RT-qPCR showed that the expression of miR-34b-5p was increased in immune system-related tissues (including the liver, spleen, and thymus) from ALV-J-infected chickens compared to those from non-infected chickens. RT-qPCR data are expressed as the fold change relative to the control (β-actin) gene. In both panels, each bar represents the mean ± the SEM of three independent experiments. Asterisks denote statistically significant differences: ^*^, ^**^, and ^***^ indicate *P* < 0.05, *P* < 0.01, and *P* < 0.001, respectively. ALV-J, Avian leukosis virus subgroup J; miRNA, microRNA; RT-qPCR, real-time quantitative polymerase chain reaction; SEM, standard error of the mean.

ALV-J, an oncogenic retrovirus, induces several leukemia-like proliferative diseases in the hemopoietic systems of chickens. Based on our previous miRNA sequencing data (GEO database accession numbers, GSE63676), miR-34b-5p was found to be significantly (*P* = 1.62E-71) upregulated in the spleens of ALV-J-infected chickens compared to the spleens of non-infected chickens (Figure 1A). In addition, RT-qPCR involving immune system-related tissues showed that the expression of miR-34b-5p was significantly increased (*P* = 0.0036 and *P* = 0.0197) in ALV-J-infected spleens and thymuses compared to non-infected spleens and thymuses, respectively (Figure 1B). In the liver, the expression of miR-34b-5p was also increased in ALV-J-infected chickens, although the difference was not significant (*P* = 0.09). These findings demonstrate that the expression of miR-34b-5p is significantly dysregulated in ALV-J-infected chickens and it might be involved in virus invasion.

### MIR-34b-5p promotes cell proliferation and migration and modulates cell cycle progression in ALV-J-infected DF-1 cells

**Figure 2:**
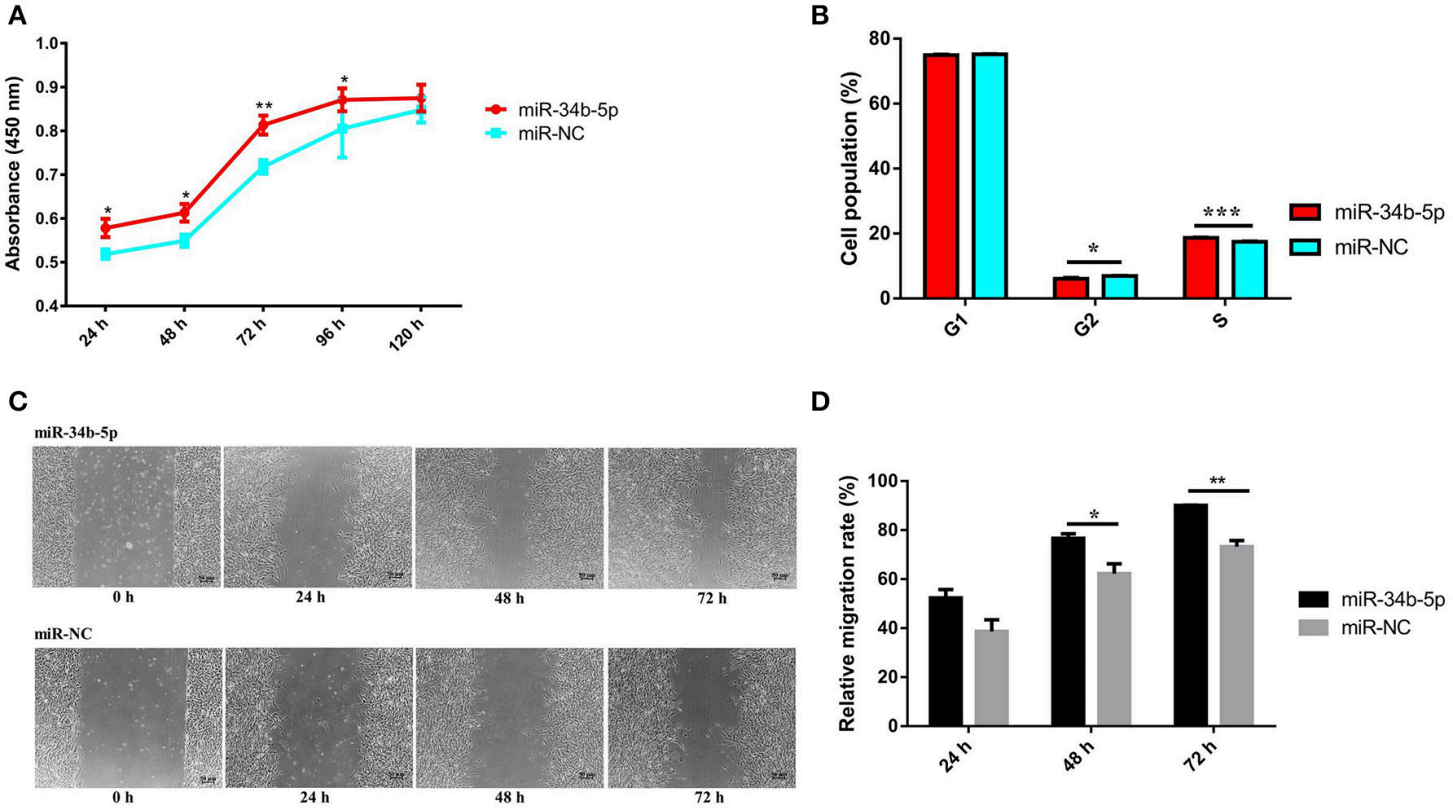
**Overexpression of miR-34b-5p promotes the proliferation and migration of ALV-J-infected DF-1 cells and it accelerates G1 to S phase transition**. Twelve hours after transfected with miR-34b-5p mimic or mi-NC, the transfected DF-1 cells were infected with ALV-J at TCID_50_. **(A)** Cell growth was significantly increased after miR-34b-5p mimic transfection compared to the control cells (*n* = 6). **(B)** Overexpression of miR-34b-5p resulted in an increased proportion of cells in the S phase and a decreased proportion of cells in the G2 phase. **(C)** Representative images of the wound healing assay at 0, 24, 48, and 72 h after ALV-J infection in miR-34b-5p mimic or mi-NC transfected DF-1 cell. **(D)** Quantification of wound healing assay at 24, 48, and 72 h after ALV-J infection. In panel **A**, the results were confirmed by three independent experiments with six (cell number) samples per treatment. In panels **(B–D)**, each bar represents the mean ± the SEM of three independent experiments. Asterisks denote statistically significant differences: ^*^, ^**^, and ^***^ indicate *P* < 0.05, *P* < 0.01, and *P* < 0.001, respectively. ALV-J, Avian leukosis virus subgroup J; mi-NC, mimic-negative control (empty plasmid); UTR, untranslated region; SEM, standard error of the mean; TCID_50_, 50% tissue culture infectious dose.

Previous studies demonstrated that miRNAs can affect various aspects of cancer biology, including cell proliferation, migration, and apoptosis (Zhao et al., 2014; Shi W. et al., 2016). To explore the role of miR-34b-5p on ALV-J infection, we investigated its influence on cellular processes in ALV-J-infected cells. Firstly, we transiently transfected an miR-34b-5p mimic or a negative control mimic (empty plasmid; miR-NC) into DF-1 cells. After 12 h in culture media, the transfected DF-1 cells were infected with ALV-J, and then cell proliferation and migration analyses were carried out. The result of the CCK-8 assay showed that overexpression of miR-34b-5p could significantly (at *P* < 0.05 or *P* < 0.01) promote the proliferation of ALV-J-infected cells compared to miR-34b-5p negative control (miR-NC) at 24~96 h after the ALV-J infection (Figure 2A). Moreover, using flow cytometry, we found that overexpression of miR-34b-5p significantly increased the number of cells in S phase (*P* < 0.001) and reduced the number of cells in G2 phase (*P* < 0.05) compared to the negative control group (Figure 2B).

Subsequently, to assess the effect of miR-34b-5p overexpression on the cell migration, a wound healing assay was performed in ALV-J-infected DF-1 cells. We found that the migration rate of DF-1 cells transfected with miR-34b-5p mimics was significantly increased (*P* < 0.05 and *P* < 0.01 at 48 and 72 h after the ALV-J infection, respectively) compared to the negative control group (Figures 2C,D). Taken together, our data suggested that miR-34b-5p promotes the growth of ALV-J-infected DF-1 cells by accelerating their proliferation and migration.

### *MDA5* is a direct target of miR-34b-5p

**Figure 3:**
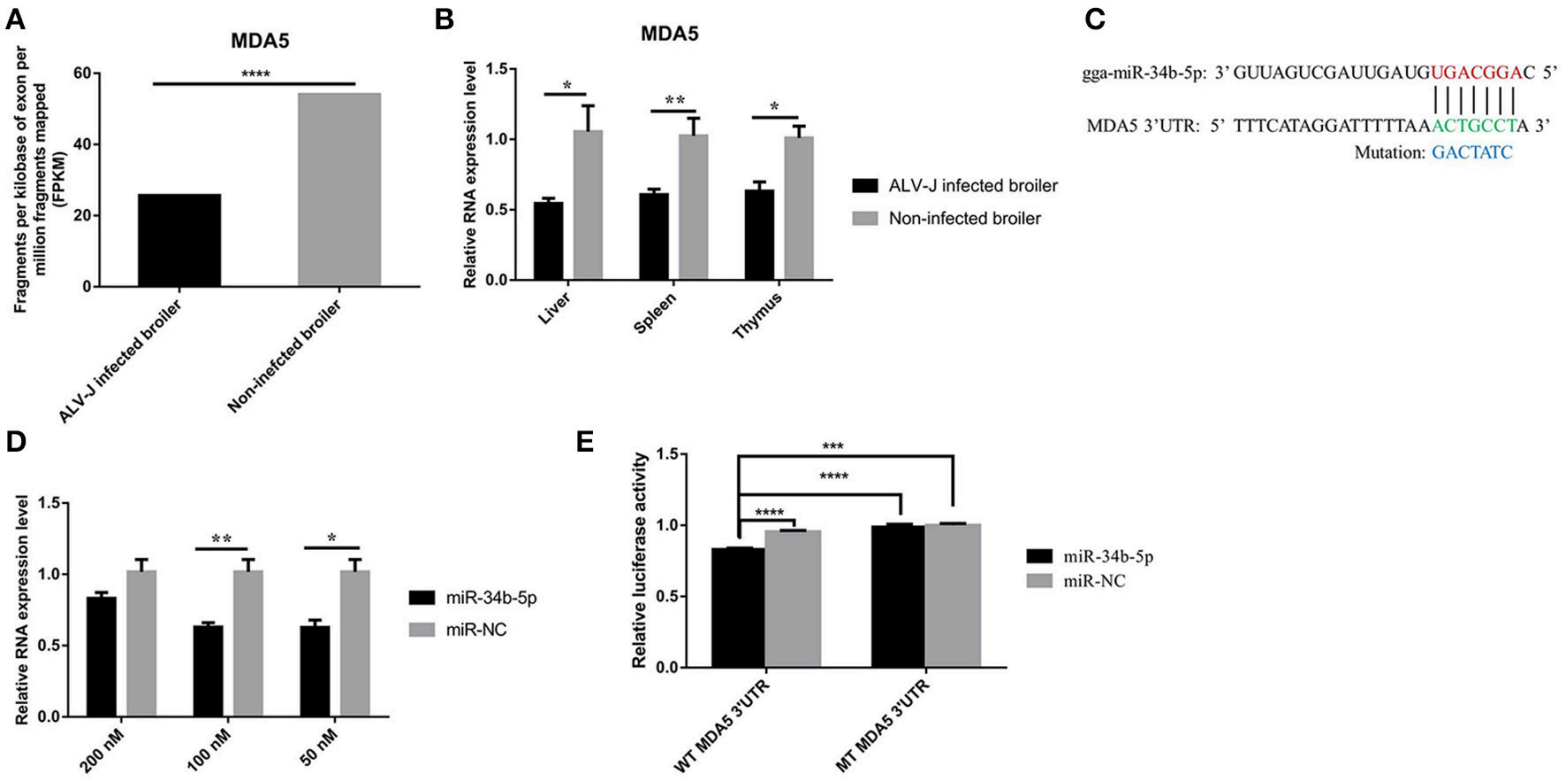
**MDA5 is a direct target of miR-34b-5p. (A)** RNA sequencing showed that the expression of *MDA5* was downregulated in the spleens of ALV-J-infected chickens. **(B)** RT-qPCR showed that the expression of *MDA5* was decreased in immune system-related tissues (including the liver, spleen, and thymus) from ALV-J-infected chickens compared to those from non-infected chickens. **(C)** MiR-34b-5p binding site in the 3′ UTR of chicken *MDA5* mRNA (in green). The mutation sequence in the miR-34b-5p binding site is highlighted in blue. **(D)** 100 and 50 nM miR-34b-5p mimic could significantly decrease the expression of *MDA5* mRNA in DF-1 cells at 48 h after transfection. **(E)** The luciferase assay was conducted by cotransfecting wildtype or mutant *MDA5* 3′ UTR with 100 nM miR-34b-5p mimic or mi-NC into DF-1 cells. Firefly luciferase activity for each sample was normalized to Renilla luciferase activity. In all panels, the data shown are the mean ± the SEM of three independent experiments. Asterisks denote statistically significant differences: ^*^, ^**^, ^***^, and ^****^ indicate *P* < 0.05, *P* < 0.01, *P* < 0.001, and *P* < 0.0001, respectively. ALV-J, Avian leukosis virus subgroup J; *MDA5*, melanoma differentiation-associated gene 5; mi-NC, mimic-negative control (empty plasmid); UTR, untranslated region; RT-qPCR, real-time quantitative polymerase chain reaction; SEM: standard error of the mean.

Our previous RNA sequencing data (GSE63226) demonstrated that *MDA5* was significantly downregulated (*P* = 1.34E-06) in the spleens of ALV-J-infected chickens compared to non-infected chickens (Figure 3A). The RT-qPCR results revealed that the expression of *MDA5* was significantly downregulated in immune system-related tissues including the livers (*P* = 0.046), spleens (*P* = 0.0022), and thymuses (*P* = 0.022) of ALV-J-infected chickens compared to those from non-infected chickens (Figure 3B). In addition, miR-34b-5p had a contrasting expression pattern in ALV-J-infected chickens compared to non-infected chickens (Figures 1, 3A,B). Using miRNA target prediction software, miRDB (http://www.mirdb.org/miRDB) (Wong and Wang, 2015), *MDA5* was predicted to be a potential target gene for miR-34b-5p. The miR-34b-5p mature sequence and its binding seed sequence in *MDA5* are shown in Figure 3C. To confirm whether miR-34b-5p directly targets the 3′ UTR of *MDA5*, a dual-luciferase reporter gene assay was carried out in DF-1 cells. The transfection efficiency analysis showed that 50 and 100 nM of an miR-34b-5p mimic could significantly (*P* = 0.019) and highly significantly (*P* = 0.0027) decrease the *MDA5* expression levels in DF-1 cells at 48 h after transfection, respectively (Figure 3D). Therefore, we co-transfected 100 nM miR-34b-5p mimic or mi-NC into DF-1 cells with reporter vectors. The dual luciferase assay showed that the wild-type group (WT-*MDA5*-3′UTR) induced a significant decrease (*P* < 0.05) in luciferase activity compared to the control group, whereas no significant difference was observed between the mutant-type group (MT-*MDA5*-3′UTR) and control group (Figure 3E). Taken together, these results suggested that *MDA5* is a direct target of miR-34b-5p.

### *MDA5* mediates the effect of miR-34b-5p on cell proliferation and migration

**Figure 4:**
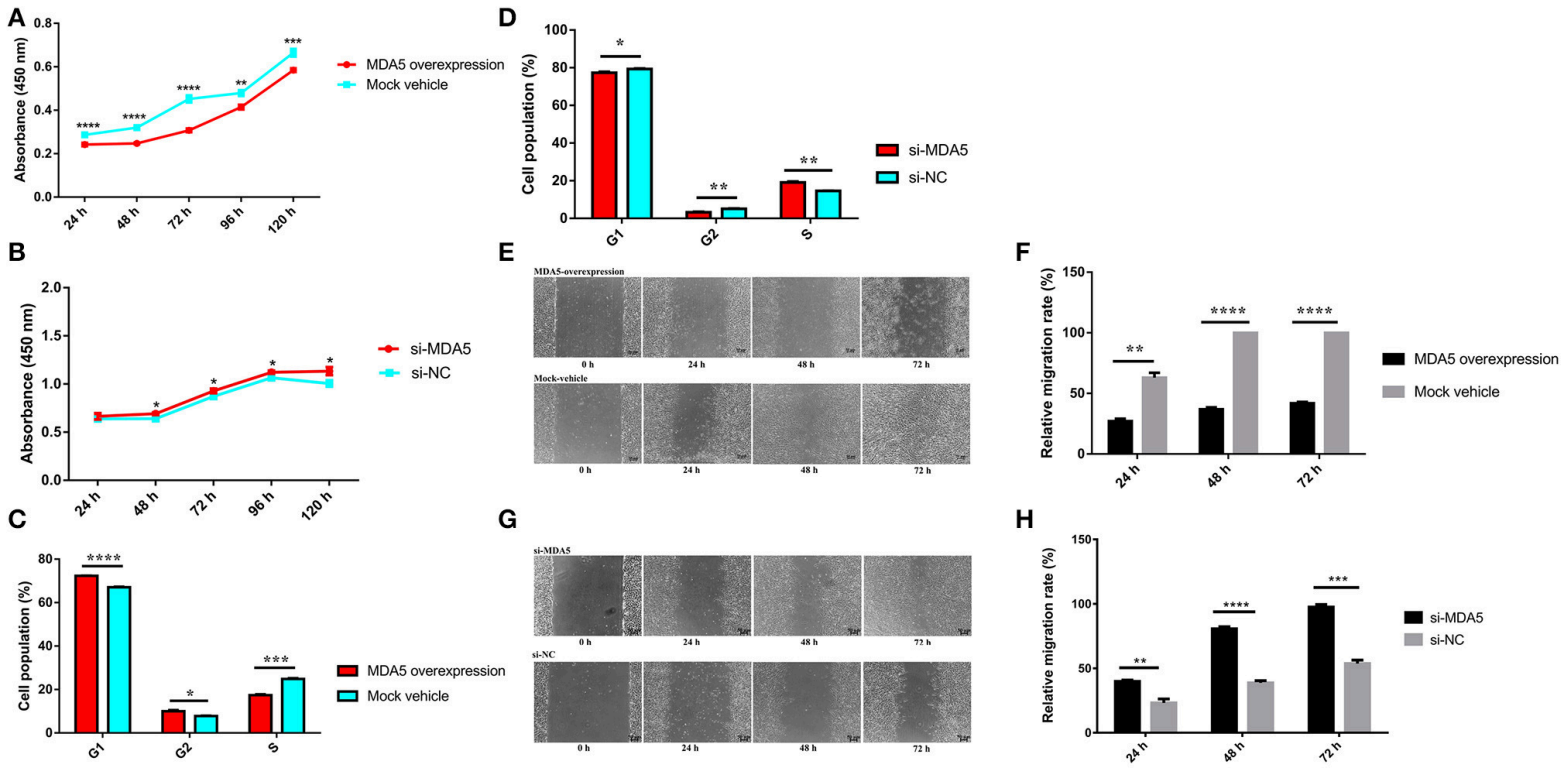
**MDA5 mediates the effects of miR-34b-5p on the proliferation, cell cycle, and migration of ALV-J-infected DF-1 cells. (A)** Cell growth was significantly reduced after the transfection of an *MDA5* overexpression plasmid compared to control cells (*n* = 6). **(B)** Cell growth was significantly increased after the transfection of a siRNA targeted against the *MDA5* gene compared to control cells (*n* = 6). **(C)** Overexpression of *MDA5* resulted in increased proportions of cells in the G1 and G2 phases and a decreased proportion of cells in the S phase. **(D)** Knockdown of *MDA5* resulted in an increased proportion of cells in the S phase and decreased proportions of cells in the G1 and G2 phases. **(E)** Representative images of the wound healing assay at 0, 24, 48, and 72 h after the ALV-J infections with *MDA5* overexpression in DF-1 cells. **(F)** Quantification of the wound healing assay of *MDA5* overexpression at 24, 48, and 72 h after the ALV-J infections. **(G)** Representative images of the wound healing assay at 0, 24, 48, and 72 h after the ALV-J infections in *MDA5* knockdown DF-1 cells. **(H)** Quantification of the wound healing assay in *MDA5* knockdown DF-1 cells. In panels **(A,B)**, the results were confirmed by three independent experiments with six (cell number) samples per treatment. In panels **(C,D,F,H)**, the data shown are the mean ± the SEM from three independent experiments. Asterisks denote statistically significant differences: ^*^, ^**^, ^***^, and ^****^ indicate *P* < 0.05, *P* < 0.01, *P* < 0.001, and *P* < 0.0001, respectively. ALV-J, Avian leukosis virus subgroup J; *MDA5*, melanoma differentiation-associated gene 5; SEM, standard error of the mean; siRNA, small interfering RNA.

To explore whether the effect of miR-34b-5p on cell processes in ALV-J-infected cells was mediated by *MDA5*, we performed CCK-8, cell cycle and wound healing assays in *MDA5* overexpression and knockdown DF-1 cells. Overexpression of *MDA5* significantly inhibited (*P* ≤ 0.01) cell proliferation compared to the control group at 24–120 h after the ALV-J infection (Figure 4A). However, *MDA5* knockdown significantly promoted (*P* ≤ 0.05) cell proliferation at 48–120 h after the ALV-J infection (Figure 4B). On the other hand, *MDA5* overexpression significantly (*P* < 0.001) decreased the proportion of ALV-J-infected cells at S phase but notably increased the proportion of cells at G1 phase (*P* < 0.0001) and G2 phase (*P* < 0.05) compared to the control group (Figure 4C). In contrast, knockdown of *MDA5* using siRNA led to a significant (*P* < 0.01) elevation in the proportion of cells at S phase, and a clear reduction in the proportion of cells at G1 phase (*P* < 0.05) or G2 phase (*P* < 0.01; Figure 4D). Moreover, the migration rate of DF-1 cells that overexpressed *MDA5* was significantly decreased (*P* ≤ 0.01) compared to that in the control group (Figures 4E,F). In contrast, the migration rate of *MDA5* knockdown cells was significantly increased (*P* ≤ 0.01) compared to those transfected with the small interfering RNA negative control (si-NC; Figures 4G,H). These results indicated that miR-34b-5p might modulate the proliferation, migration, and cell cycle of ALV-J-infected DF-1 cells by targeting *MDA5*.

### *MDA5* can detect ALV-J infections and trigger the MDA5 signaling pathway

**Figure 5:**
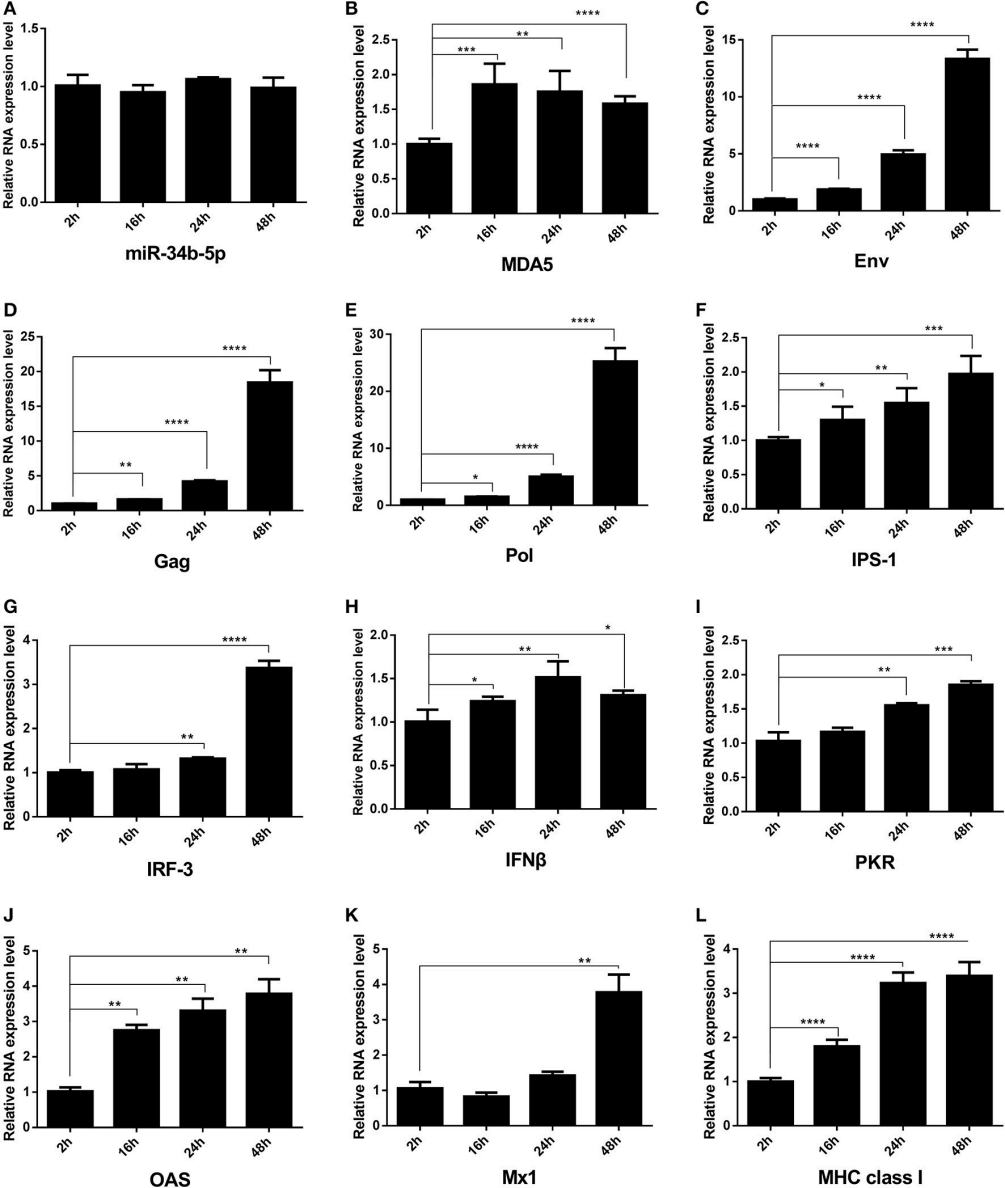
**The expression of mRNA of ALV-J ***env gag, pol*** and MDA5 signaling pathway-related innate and antiviral genes in chicken DF-1 cells during ALV-J infections**. DF-1 cells were infected with ALV-J at TCID_50_. The change in the expression of **(A)** miR-34b-5p, **(B)** *MDA5*, **(C)** *Env*, **(D)** *gag*, **(E)** *Pol*, **(F)** *IPS-1*, **(G)** *IRF-3*, **(H)** *IFN*β, **(I)** *PKR*, **(J)** *OAS*, **(K)** *Mx1*, and **(L)** *MHC class I* in ALV-J-infected DF-1 cells from 2 to 48 h after the infection. In all panels, each bar represents the mean ± the SEM of three independent experiments. Asterisks denote statistically significant differences: ^*^, ^**^, ^***^, and ^****^ indicate *P* < 0.05, *P* < 0.01, *P* < 0.001, and *P* < 0.0001, respectively. ALV-J, Avian leukosis virus subgroup J; *IFN*β, interferon-β; *IPS*-1, interferon-β promoter stimulator 1; *IRF-3*, interferon regulatory factor-3; *MDA5*, melanoma differentiation-associated gene 5; *MHC*, major histocompatibility complex; *Mx1*, myxovirus (influenza virus) resistance 1, interferon-inducible protein p78 (mouse); *OAS*, 2′, 5′-oligoadenylate synthetase; *PKR*, double-stranded RNA-dependent protein kinase; SEM, standard error of the mean; TCID_50_, 50% tissue culture infectious dose.

The ALV RNA genome has a typical slowly-transforming replication-competent structure: 5′ R-U5-*gag-pol-env*-U3-R 3′ that contains three coding genes: *Gag, Pol*, and *Env*. The *env* gene codes for an envelope glycoprotein that determines subgroup specificity, neutralization, and receptor binding (Pan et al., 2012). When miR-34b-5p was normal expression (Figure 5A), the expression level of *MDA5* was upregulated (Figure 5B) at 16, 24, and 48 h after infection compared to at 2 h after infection. In DF-1 cells infected with ALV-J, we found that the expression of the *Env, Gag and Pol* mRNA was significantly upregulated at 16, 24, and 48 h after infection compared to at 2 h after infection (*P* < 0.05; Figures 5C–E). To investigate whether chicken MDA5 signaling pathway was able to detect ALV-J infections and then to initiate and amplify an innate immune response, RT-qPCR was used to measure the changes in the expression of innate and antiviral genes in the MDA5 signaling pathway during ALV-J infections. The results showed that the levels of all the mRNA, including those associated with *IPS-1, IRF-3, IFN*β*, PKR, OAS, Mx1*, and *MHC class I* were upregulated at 24 and 48 h after infection compared to at 2 h after infection (Figures 5F–L). In contrast, after overexpression of miR-34b-5p by miRNA mimic (Supplementary Figure 1A) and following inoculation with TCID_50_ of ALV-J, the expression level of *MDA5* was downregulated (Supplementary Figure 1B) in miR-34b-5p overexpression DF-1 cells compared with negative control (NC) group at 2-48 h ALV-J infection time points. In addition, the mRNA expression of ALV-J related genes, including *Env, Gag* and *Pol* (Supplementary Figures 1C–E, respectively) were upregulated in miR-34b-5p overexpression DF-1 cells compared with NC group during at 2–48 h ALV-J infection time points. Moreover, the mRNA expression of MDA5 signaling pathway-related innate and antiviral genes (*IPS-1, IRF-3, IFNB, PKR, OAS, MX1, MHC class 1*) were downregulated compared with control (Supplementary Figures 1F–L). These findings indicate that miR-34b-5p suppresses MDA5 signaling pathway to promote ALV-J replication.

**Figure 6:**
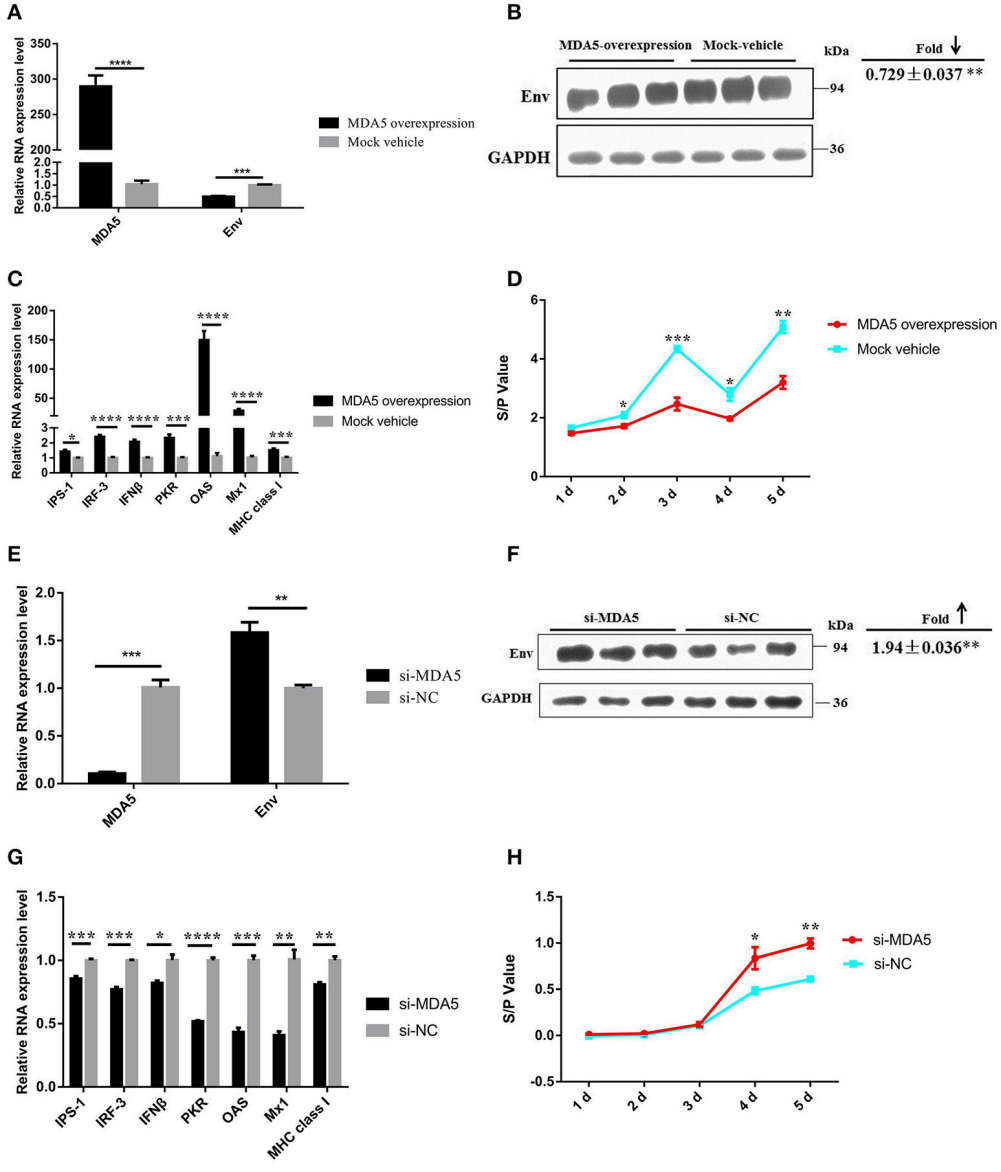
*****MDA5*** can detect ALV-J infections and trigger the MDA5 signaling pathway**. The expression of ALV-J *env* **(A)** mRNA and **(B)** protein were downregulated in DF-1 cells that overexpressed *MDA5* 3 days after the ALV-J infections. **(C)** The mRNA expression of MDA5 signaling pathway-related innate and antiviral genes was upregulated in DF-1 cells that overexpressed *MDA5*. **(D)** The sample-to-positive ratio of virion secreted in the supernatants was downregulated in DF-1 cells that overexpressed *MDA5* (*n* = 6). The expression of ALV-J *env* (**E**) mRNA and (**F**) protein were upregulated in *MDA5* knockdown DF-1 cells. **(G)** The mRNA expression of MDA5 signaling pathway-related innate and antiviral genes was downregulated in *MDA5* knockdown DF-1 cells. **(H)** The sample-to-positive ratio of virion secreted in the supernatants was upregulated after *MDA5* knockdown by siRNA (*n* = 6). In panels **(A–C,E–G)**, the data shown are the mean ± the SEM from three independent experiments. In panels **(D,H)**, the results were confirmed by three independent experiments with six (cell number) samples per treatment. Asterisks denote statistically significant differences: ^*^, ^**^, ^***^, and ^****^ indicate *P* < 0.05, *P* < 0.01, *P* < 0.001, and *P* < 0.0001, respectively. ALV-J, Avian leukosis virus subgroup J; *MDA5*, melanoma differentiation-associated gene 5; SEM, standard error of the mean; siRNA, small interfering RNA.

To further elucidate the function of *MDA5* on the MDA5 signaling pathway and ALV-J replication, ectopic expression, and knockdown of *MDA5* was carried out in DF-1 cells. In DF-1 cells that overexpressed *MDA5*, the mRNA and protein expression of *Env* and the ALV-J virion secretion were significantly suppressed (*P* ≤ 0.05) and the mRNA expression of genes in the MDA5 signaling pathway was significantly upregulated (*P* ≤ 0.05; Figures 6A–D). In contrast, after knockdown of *MDA5* by siRNA, the mRNA and protein expression of *env* were upregulated (*P* < 0.05; Figures 6E,F). The expression of genes in the MDA5 signaling pathway was significantly downregulated (*P* ≤ 0.05), and sample-to-positive ratios of virion secretion in the supernatants were elevated at 4 and 5 days after the infection (*P* ≤ 0.05; Figures 6G,H). These data suggest that *MDA5* can recognize ALV-J infections and initiate and amplify an innate immune response via the MDA5 signaling pathway in chickens.

### MIR-34b-5p modulates the MDA5 signaling pathway and promotes ALV-J replication

**Figure 7:**
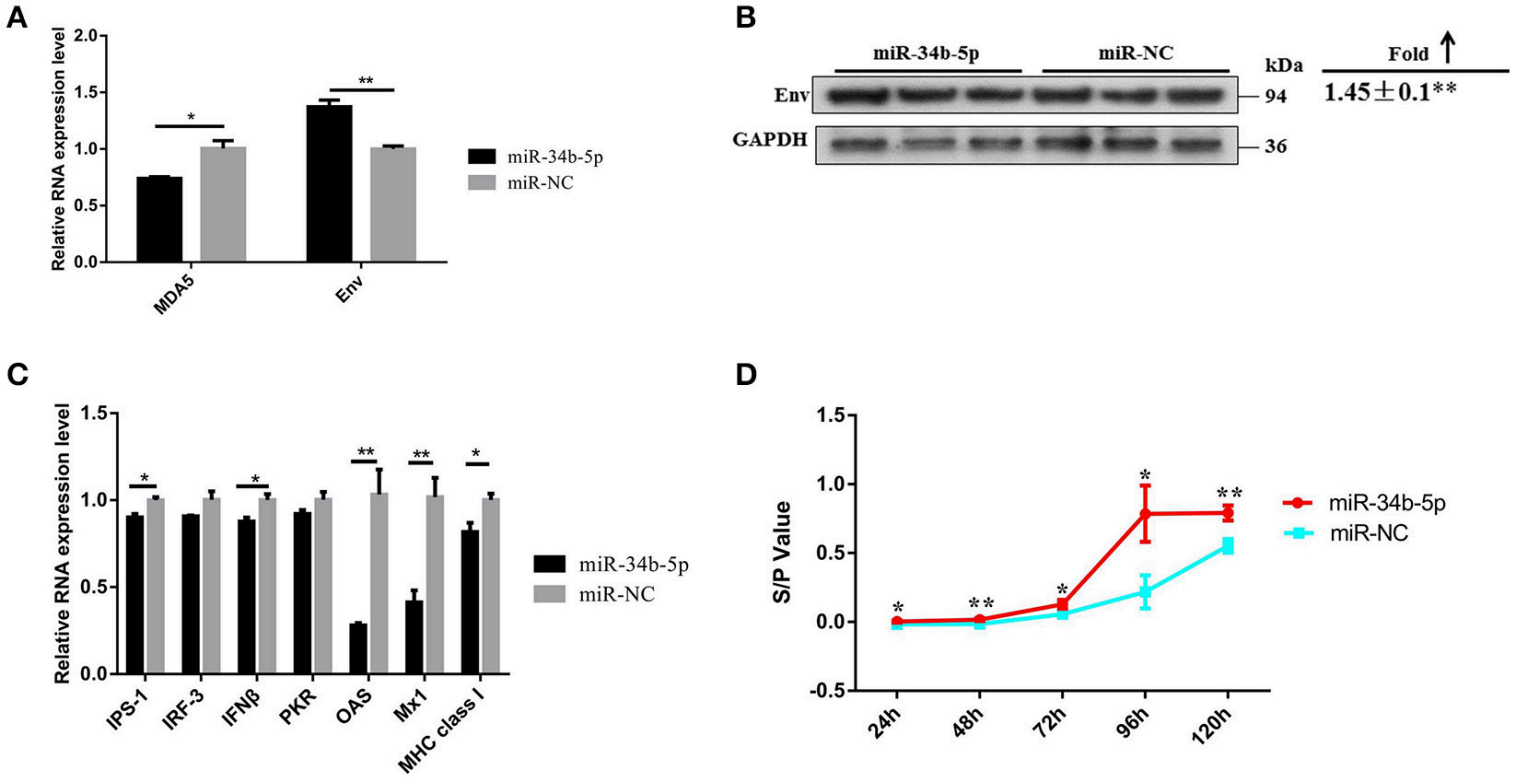
**Overexpression of miR-34b-5p suppresses the MDA5 signaling pathway and promotes ALV-J replication**. The **(A)** mRNA and **(B)** protein expression level of *env* were upregulated in DF-1 that overexpressed miR-34b-5p cells 3 days after the ALV-J infections. **(C)** The expression of mRNA of MDA5 signaling pathway-related innate and antiviral genes was downregulated in DF-1 cells that overexpressed miR-34b-5p. **(D)** The sample-to-positive ratio of virion secreted in supernatants was upregulated in DF-1 cells that overexpressed miR-34b-5p (*n* = 6). In panels **(A–C)**, the values are expressed as the mean ± the SEM of three independent experiments. In panel **(D)**, the results were confirmed by three independent experiments with six (cell number) samples per treatment. Asterisks denote statistically significant differences: ^*^ and ^**^ indicate *P* < 0.05 and *P* < 0.01, respectively. ALV-J, Avian leukosis virus subgroup J; *MDA5*, melanoma differentiation-associated gene 5; SEM, standard error of the mean.

As our findings suggested that miR-34b-5p might promote the proliferation and migration of ALV-J-infected DF-1 cells by targeting *MDA5*, we assessed the effects of miR-34b-5p overexpression on the MDA5 signaling pathway to explore the mechanism by which miR-34b-5p contributes to ALV-J infections. In DF-1 cells that overexpressed miR-34b-5p, the mRNA and protein expression level of *Env* were significantly elevated 3 days after the infection (*P* < 0.01; Figures 7A,B). Moreover, ectopic expression of miR-34b-5p significantly inhibited the expression of genes in the MDA5 signaling pathway, including *MDA5, IPS-1, IFN*β, *OAS, Mx1*, and *MHC class I* (*P* ≤ 0.05), in ALV-J-infected DF-1 cells (Figures 7A,C). The sample-to-positive ratio of virion secreted in the supernatants was also significantly upregulated (*P* ≤ 0.05) in DF-1 cells that overexpressed miR-34b-5p during ALV-J infections (Figure 7D). These data indicate that miR-34b-5p targets *MDA5* to modulate the MDA5 signaling pathway and further promote ALV-J replication.

## Discussion

We found that the expression of miR-34b-5p was increased in ALV-J-infected tissues and that ectopic expression of miR-34b-5p accelerated the proliferation and migration of ALV-J-infected cells by targeting *MDA5. In vitro* experiments revealed that the MDA5 signaling pathway, ALV-J related gene expression, and virion secretion can be regulated by miR-34b-5p. Accumulated evidence has indicated that miRNAs are involved in tumor development, and aberrant miRNA expression is closely associated with tumorigenesis (Zhang et al., 2007; Asangani et al., 2008; Lovat et al., 2011; Tomasetti et al., 2016). To date, several miRNAs, including gga-miR-221, gga-miR-222, gga-miR-23b, gga-miR-375, gga-miR-125b, gga-miR-1650, gga-miR-193a, gga-miR-193b, gga-let-7b, gga-let-7i, gga-miR-458, gga-miR-1456, gga-miR-1704, gga-miR-1777, gga-miR-1790, and gga-miR-2127, have been reported to be associated with tumorigenesis and the aberrant expression of the retrovirus, ALV-J (Li et al., 2012; Wang et al., 2013a,b; Li H. et al., 2014; Dai et al., 2015; Li et al., 2015). Our previous miRNA sequencing data indicated that miR-34b-5p expression was increased in ALV-J-infected spleens. We confirmed these results in the present study and discovered that miR-34b-5p also was upregulated in other immune system-related tissues.

MiR-34b-5p is a member of the miR-34 family. Previous studies have suggested that members of the miR-34 family may act as tumor suppressors (He et al., 2007; Hermeking, 2010). However, most previous studies have been focused on two other members of this family, miR-34a and miR-34c. It has been well-documented that miR-34b-5p is involved in the regulation of spermatogenesis (Smorag et al., 2012; Sakurai et al., 2015). In addition, the miR-34b-5p/Notch1 pathway was reported to be important for the oncogenesis involved in minimal deviation adenocarcinoma (Lee H. et al., 2014). To our knowledge, this study is the first study to demonstrate that miR-34b-5p may be involved in the pathogenesis of ALV-J, as it appears to affect many aspects of cancer biology, such as cell proliferationand cell cycle progression. We further investigated the biological functions of miR-34b-5p using an overexpression experiment (Zhao et al., 2015; Shi B. et al., 2016). The functional investigation demonstrated that ectopic expression of miR-34b-5p could accelerate cell proliferation and migration in ALV-J-infected cells. The cell cycle comprises three phases: G1, which involves preparation for DNA synthesis; S, which involves DNA synthesis; and G2, which involves preparation for mitosis (Zhang et al., 2009). We found that miR-34b-5p overexpression increased the proportion of cells in S phase and reduced the proportion of cells in G2 phase, suggesting that it can induce cells from the G1 to the S phase.

It is well-known that each miRNA can regulate up to hundreds of target genes (Lewis et al., 2005). The use of prediction software revealed that miR-34b-5p may have an effect on a large number of target genes. Among these, three oncogenes, *Notch1, c-Met*, and *CDK6*, have been confirmed to be targets of miR-34b-5p (Smorag et al., 2012; Lee C. C. et al., 2014; Li et al., 2016). We predicted that the *MDA5* gene was a target of miR-34b-5p using miRDB software and integrative analyses of our previous RNA and small RNA sequencing data (GEO database, accession numbers, GSE63226 and GSE63676, respectively). Subsequently, we confirmed this prediction using RT-qPCR and a dual-luciferase reporter assay.

The PRRs of host innate immune system, including Toll-like receptors (TLRs), retinoic acid-inducible gene I-like helicases (RLHs), and nucleotide-oligomerization domain-like receptors (NLRs), can detect viral infections and evoke antiviral responses by producing type I IFNs. *MDA5* belongs to the RLH family and it is able to detect viral RNA in the cytoplasm (Pichlmair et al., 2006). The domain architecture of chicken *MDA5* comprises two caspase recruitment domains (CARDs), a restriction enzyme domain type 3 (RES III), a helicase conserved C-terminal domain (helicase-C), and a retinoic acid-inducible gene I C-terminal regulatory domain (RIG-I-C-RD) (Lee et al., 2012) During a viral infection, MDA5 detects viral RNA that has a helicase domain and transmits a signal via CARDs. The activated CARDs associate with an adaptor protein, *IPS-1*, and this leads to the recruitment and activation of both canonical and non-canonical inhibitors of NF-κB kinase (IKK) complexes. Furthermore, the activated IKK complexes mediate the phosphorylation of IRF-3, which induces the formation of homodimers and/or heterodimers. The functional IRF dimers translocate to the cell nucleus and interact with IFN-stimulated response elements (ISREs), which leads to the transcription of *IFN*β and a set of IFN-inducible genes (*PKR, OAS, Mx1*, and *MHC class I*; Kato et al., 2006; Takeuchi and Akira, 2008; Karpala et al., 2011).

Our results showed that in the natural infection in tissues of broilers caused by ALV-J, the expression level of miR-34b-5p was aberrantly upregulated (Figures 1A,B); whereas, the expression level of its target gene *MDA5* was significantly down-regulated (Figures 3A,B). On the other hand, in DF-1 cells infected ALV-J, when miR-34b-5p was normally expressed (Figure 5A), the expression level of MDA5 was up-regulated at 16 h, 24 h, 48 h compared with 2 h after ALV-J infection (Figure 5B). These differences in the expression levels could possibly be due to the subject of time after ALV-J infection between the tissues (spleen) and DF-1 cells i.e., the time of spleen-infected broiler was longer (140-day-old spontaneous infectious broilers) than those in DF-1 cells infected for not more than 48 h. It has been reported that the ALV-J viral stock could cause fibroblastic sarcoma in chickens within 14 days but gave rise to a moderate transformation in the culture of primary chicken embryonic fibroblast cells (Wang et al., 2015). In order to confirm the assumption above, we carried out miR-34b-5p mimic transfection in DF-1 and then infected with ALV-J, after miR-34b-5p mimic transfection, the RNA expression level of miR-34b-5p was significantly upregulated than that in NC group (Supplementary Figure 1A). In addition, when miR-34b-5p was overexpressed, the expression level of *MDA5* was downregulated than that in NC group (Supplementary Figure 1B). These results (ALV-J-infected DF-1 cells with miR-34b-5p overexpression; Supplementary Figure 1A,B) are consistent with natural ALV-J infection broilers (Figures 1, 3A,B). Furthermore, we investigated the mRNA expression level of ALV-J-related genes after miR-34b-5p overexpressed in DF-1 cells; the mRNA expression level of Env (Supplementary Figure 1C), Gag (Supplementary Figure 1D) and Pol (Supplementary Figure 1E) were upregulated in ALV-J-infected DF-1 cells compared with that in NC group at 2–48 h ALV-J infection time points. Taken together, these data suggest that miR-34b-5p could inhibit *MDA5* to promote ALV-J replication in DF-1 cells.

**Figure 8:**
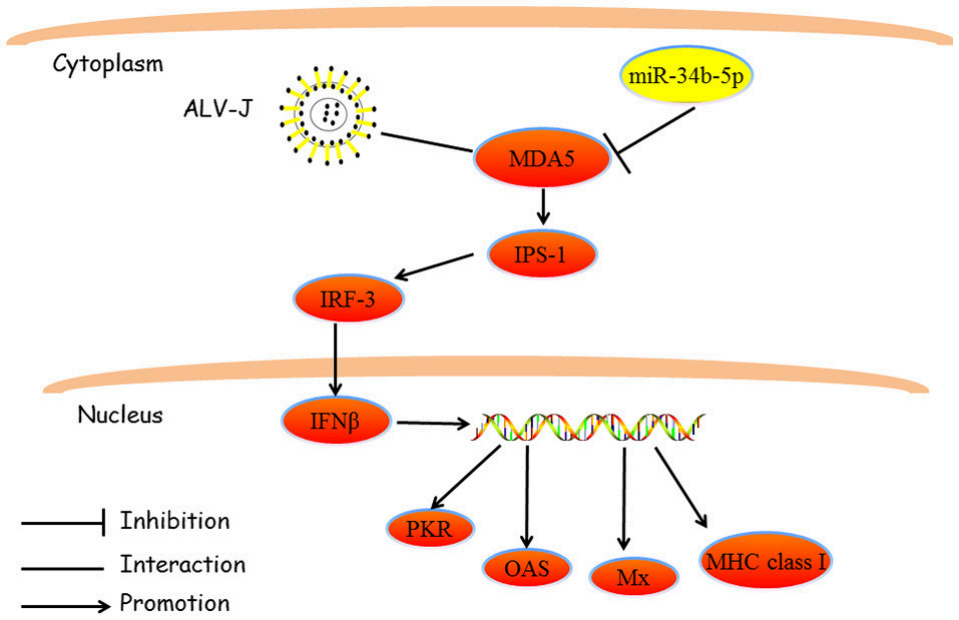
**A schematic representation showing the miR-34b-5p-mediated MDA5 signaling pathway during ALV-J infections**. This model is based on knowledge of the MDA5-mediated signaling pathway. As a member of the RLH family, *MDA5* can detect ALV-J infections in the cytoplasm. Abnormal expression of miR-34b-5p suppressed the function of *MDA5*, which triggered the signal transduction cascade to induce an IFNβ response and which, in turn, upregulated downstream antiviral genes (*PKR, OAS, Mx1*, and *MHC class I*). MiR-34b-5p inhibited the expression of *MDA5*, and led to increased proliferation and migration of the ALV-J-infected cells, and promoted ALV-J replication. ALV-J, Avian leukosis virus subgroup J; *IFN*β, interferon-β; *MDA5*, melanoma differentiation-associated gene 5; *MHC*, major histocompatibility complex; *Mx1*, myxovirus (influenza virus) resistance 1, interferon-inducible protein p78 (mouse); *OAS*, 2′, 5′-oligoadenylate synthetase; *PKR*, double-stranded RNA-dependent protein kinase; RLH, retinoic acid-inducible gene I-like helicases.

Moreover, we found that *MDA5* plays an important role in controlling the proliferation, cell cycle progression, and migration of ALV-J-infected cells (Figure 4). Recently, antiviral signaling pathways have attracted a great deal of attention from researchers and there are many studies on the cellular pathways involved in ALV-J infections. This research is necessary to develop methods to control viral infections and/or help to devise treatments for the infections. At present, several pathways have been reported to play important roles in ALV-J replication and infection, including the phosphoinositide 3-kinase (PI3K)/AK mouse strain thymic lymphoma gene (Akt) pathway, the ERK/ activator protein 1 (AP1) pathway, the MAPK signaling pathway, the wingless-related integration site (Wnt) signaling pathway, cytokine-cytokine receptor interaction, the Janus kinase (JAK)/signal transducers and activators of transcription (STAT) signaling pathway and the RLR signaling pathway (Feng et al., 2011; Wang et al., 2013a; Dai et al., 2016). We observed that *MDA5* can affect the MDA5 signaling pathway, and there was a clear correlation between this pathway and virion secretion. These observations strongly indicate that the MDA5 signaling pathway is involved in ALV-J replication. In this study we proposed mechanism of miR-34b-5p actions that mediated MDA5 signaling pathway during ALV-J infections (Figure 8).

In conclusion, our findings revealed that miR-34b-5p was increased in ALV-J-infected tissues and that ectopic expression of miR-34b-5p accelerated the proliferation and migration of ALV-J-infected cells and modulated cell cycle progression by targeting *MDA5*. Further *in vitro* investigations revealed that miR-34b-5p can regulate the MDA5 signaling pathway, ALV-J related gene expression, and virion secretion. These results demonstrated that miR-34b-5p targets *MDA5* to inhibit the expression of genes in the MDA5 signaling pathway (*IPS-1, IRF-3, IFN*β*, PKR, OAS, Mx1*, and *MHC class I*), accelerate the proliferation and migration of ALV-J-infected cells and promote ALV-J replication (Figure 8). These findings provide a solid foundation for the understanding of the underlying mechanisms of ALV-J pathogenesis and they will contribute to the development of studies on other retroviruses.

## Author contributions

ZL performed the research, analyzed the data, and wrote the paper. QL participated in the study design, analyzed the data, and wrote the paper. HX analyzed the data and wrote the paper. MZ analyzed the data. BA reviewed the manuscript. QN conceived of the study and participated in its design and coordination. XQZ participated in the design of the study. MF, BC, and XCZ contributed reagents and other resources.

## Funding

This research was supported by the Program for New Century Excellent Talents in University (NCET-13-0803), the Natural Scientific Foundation of China (31571269), the Foundation for High-level Talents in Higher Education in Guangdong, China, and the Science and Technology Research Project of Guangzhou (201508020075).

### Conflict of interest statement

The authors declare that the research was conducted in the absence of any commercial or financial relationships that could be construed as a potential conflict of interest.
