# Supplementary material for: MiR-34b-5p Suppresses Melanoma Differentiation-Associated Gene 5 (MDA5) Signaling Pathway to Promote Avian Leukosis Virus Subgroup J (ALV-J)-Infected Cells Proliferaction and ALV-J Replication
Source: Front Cell Infect Microbiol. 2017 Jan 30;7:17. doi: 10.3389/fcimb.2017.00017 (PMC5276853; doi:10.3389/fcimb.2017.00017)
Supplement: Supplementary file 1 [file DataSheet1.PDF]

Supplementary Information

MiR-34b-5p Suppresses Melanoma Differentiation-Associated Gene 5 (MDA5) Signaling Pathway to Promote Avian Leukosis Virus Subgroup J (ALV-J) Infected Cells Proliferation and ALV-J Replication

Zhenhui Li[1, 2], Qingbin Luo[1, 2], Haiping Xu[1, 2], Ming Zheng[1, 2], Bahareldin Ali Abdalla[1 ,2], Min Feng[1, 2], Bolin Cai[1, 2], Xiaocui Zhang[1, 2], Qinghua Nie[1, 2*], and Xiquan Zhang[1, 2]

Running title: MiR-34b-5p promotes ALV-J replication

[1] Department of Animal Genetics, Breeding and Reproduction, College of Animal Science, South China Agricultural University, Guangzhou, Guangdong, China

[2] Guangdong Provincial Key Lab of Agro-Animal Genomics and Molecular Breeding and the Key Lab of Chicken Genetics, Breeding and Reproduction, Ministry of Agriculture, Guangzhou, Guangdong, China

Correspondence to:

Qinghua Nie

nqinghua@scau.edu.cn

Keywords: MiR-34b-5p; melanoma differentiation associated gene 5 (MDA5);

Avian leukosis virus subgroup J (ALV-J); Cell -proliferation; MDA5 Signaling Pathway

Supplementary Figure S1: Overexpression of miR-34b-5p suppresses the *MDA5* signaling pathway and promotes ALV-J replication in DF-1 cells. DF-1 cells were seeded in 24-well plates, when the cells grew to a density of 50% confluence; they were transfected with 100 nM of gga-miR-34b-5p mimics (overexpression group) or 100 nM of mimic control duplexes (NC). 12 hours after miRNA mimic transfection, the DF-1 cells were infected with ALV-J at $TCID_{50.}$ At 2 h, 16 h, 24 h, and 48 h after ALV-J infection time points, the total RNA of DF-1 cells were isolated for qPCR analysis. **(A)** After miR-34b-5p mimic transfection, the RNA expression level of miR-34b-5p was significantly upregulated than that in the negative control (NC) group at 2-48 h ALV-J infection time points. **(B)** When miR-34b-5p was overexpressed, the expression level of *MDA5* was downregulated than that in NC group at 2-48 h ALV-J infection time points. The mRNA expression of ALV-J-related genes *Env* **(C)**, *Gag* **(D)** and *Pol* **(E)** were upregulated after miR-34b-5p overexpression in DF-1 cells compared with NC group at 2-48 h ALV-J infection time points. **(F-L)** The expression of mRNA level of MDA5 signaling pathway related innate and antiviral genes was downregulated after miR-34b-5p overexpression in DF-1 cells compared with NC group at 2-48 h ALV-J infection time points. Asterisks denote statistically significant differences: *, **, ***, and **** indicate $P < 0.05$, $P < 0.01$, $P < 0.001$ and $P < 0.0001$, respectively. SEM: standard error of the mean.

Figure S1

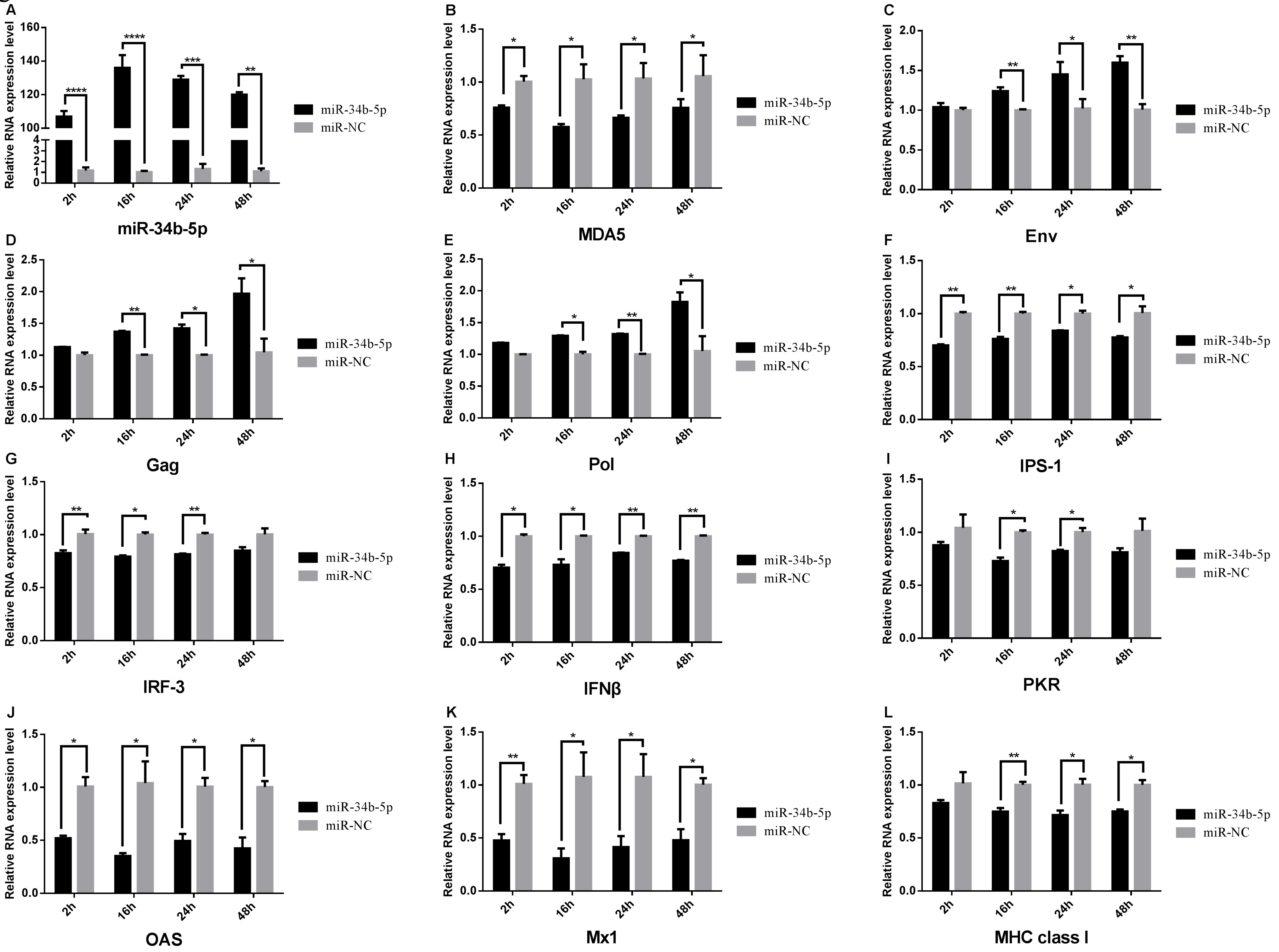

A
miR-34b-5p
B
MDA5
C
Env
D
Gag
E
Pol
F
IPS-1
G
IRF-3
H
IFNβ
I
PKR
J
OAS
K
Mx1
L
MHC class I
Relative RNA expression level
2h
16h
24h
48h
miR-34b-5p
miR-NC

Supplementary Table S1: Primers used for quantitative real-time PCR

Target	Primer	Sequence (5'-3')	Reference
MDA5	Forward	GAACCATTAGTATCAAAACA	
	Reverse	GAACTCTTCCATTAAAGTATT	
ALV-J Env	Forward	AAAACGACACCGATTTAGCC	
	Reverse	AGTCACGCAGTTCTGCTTCC	
ALV-J Gag	Forward	TTTAGGACCTGCCCCATACG	
	Reverse	TGCCCACCATCCCATCAG	
ALV-J Pol	Forward	GGGTAATTCCCAGGGTCAAG	
	Reverse	TTCTGTAAGAACGGTAGGTCTCC	
Mx1	Forward	ATCAGAGGTGAAGAAAGCAA	
	Reverse	TTCCAGGAAGATCAATTAGTGT	
IPS-1	Forward	GCAGTTTGATGCAGAGCAGAAGCA	Lee et al., 2014
	Reverse	AGGCTTCAAGGAGGTGTCACAGAA	
IRF-3	Forward	ACCACATGCAGACAGACTGACACT	Lee et al., 2014
	Reverse	GGAGTGGATGCAAATGCTGCTCTT	
IFNβ	Forward	ACCAGGATGCCAACTTCTCTTGGA	Lee et al., 2014
	Reverse	ATGGCTGCTTGCTTCTTGTCCTTG	
PKR	Forward	ACGTGGGACATGATTGAGCCAAAG	Lee et al., 2014
	Reverse	TGATGTAGTCAACTGGAGGGAGCA	
OAS	Forward	GCAGAAGAACTTTGTGAAGTGGCG	Lee et al., 2014
	Reverse	TCGGCTTCAACATCTCCTTGTACC	

MHC class I	Forward	AGTTCCCACCAAGAGGAAATGGGA	Lee et al., 2014
	Reverse	ATTCCACATATCTCCGCAGCCACT	
β-actin	Forward	GATATTGCTGCGCTCGTTG	
	Reverse	TTCAGGGTCAGGATACCTCTTT	

Supplementary Table 2: Primers used for clone of the full-length *MDA5* CDs and the 3'UTR of *MDA5*.

**Target**	**Primer**	**Sequence (5'-3')**
MDA5-CDs	Forward	GGGGggtctctagtgAGCCTGAGAACCGCTGAC
	Reverse	GCCGggtctcgtgggAGGAGCCTTCATAGTGTTTA
MDA5-3UTR	Forward	CTAgctagcGTTTTGTATTAGAGGCAC
	Reverse	ACGCgtcgacCTATTACAGTCATTTATTGG

Supplementary Table S3: Oligonucleotide sequences of si-MDA5

**siRNA name**	**Sense and antisense**	**siRNA oligonucleotide sequence**
**si-MDA5-01**	**Sense (5'-3')**	**GCAUCUCAUUGUCCUUCAA dTdT**
	**Antisense (3'-5')**	**dTdT CGUAGAGUAACAGGAAGUU**
**si-MDA5-02**	**Sense (5'-3')**	**GCUGGACACAAUAGUGAAAdTdT**
	**Antisense (3'-5')**	**dTdTCGACCUGUGUUAUCACUUU**
**si-MDA5-03**	**Sense (5'-3')**	**GGUAUCAAGUUAUUGGCUUdTdT**
	**Antisense (3'-5')**	**dTdTCCAUAGUUCAAUAACCGAA**
**si-MDA5-04**	**Sense (5'-3')**	**GCAGAACACUUGAAGAAAUdTdT**

	Antisense (3’-5’)	dTdTCGUCUUGUGAACUUCUUUA

Supplementary Table S4: The length of the scratch in wound healing assay

**Treatment**	**Repetition**	**The width of scratch at different time points**			
		**0 h (μm)**	**24 h (μm)**	**48 h (μm)**	**72 h (μm)**
**MiR-34b-5p**	**01**	**993.16**	**452.14**	**235.83**	**99.15**
	**02**	**1010.26**	**561.54**	**274.36**	**102.50**
	**03**	**962.31**	**401.71**	**183.76**	**92.50**
**MiR-NC**	**04**	**1026.50**	**509.40**	**349.57**	**289.75**
	**05**	**1018.80**	**688.89**	**323.08**	**212.82**
	**06**	**1021.37**	**680.36**	**484.62**	**315.39**
**MDA5 overexpression**	**07**	**1087.72**	**805.98**	**721.36**	**661.54**
	**08**	**1047.02**	**796.39**	**662.39**	**601.72**
	**09**	**1054.39**	**717.86**	**627.35**	**590.60**
**Mock-vehicle**	**10**	**1069.23**	**315.39**	**0**	**0**
	**11**	**1042.74**	**482.92**	**0**	**0**
	**12**	**1017.10**	**357.27**	**0**	**0**
**Si-MDA5**	**13**	**1076.07**	**661.40**	**169.23**	**0**
	**14**	**1226.32**	**707.69**	**243.59**	**0**
	**15**	**1201.76**	**733.33**	**269.23**	**88.61**
**Si-NC**	**16**	**1272.81**	**907.50**	**729.06**	**638.60**
	**17**	**1206.67**	**1010.53**	**749.12**	**480.70**

	**18**	**1236.75**	**930.77**	**794.87**	**602.57**

Supplemental references

1. Lee, C. C., Wu, C. C., and Lin, T. L. (2014). Chicken melanoma differentiation-associated gene 5 (MDA5) recognizes infectious bursal disease virus infection and triggers MDA5-related innate immunity. *Arch Virol*. 159: 1671-86. doi: 10.1007/s00705-014-1983-9
